# COVID-19 infection control measures and outcomes in urban dialysis centers in predominantly African American communities

**DOI:** 10.1186/s12882-021-02281-6

**Published:** 2021-03-06

**Authors:** Ibironke W. Apata, Jason Cobb, Jose Navarrete, John Burkart, Laura Plantinga, Janice P. Lea

**Affiliations:** 1 Division of Renal Medicine, Emory University School of Medicine, 101 Woodruff Circle, WMB 3300, Atlanta, GA 30322 USA; 2 Renal Division, Wake Forest University School of Medicine, Winston-Salem, NC USA; 3 Division of Geriatrics and Gerontology, Emory University School of Medicine, Atlanta, GA USA

**Keywords:** Dialysis, COVID-19, SARS-CoV-2, Infection control

## Abstract

**Background:**

Emory Dialysis serves an urban and predominantly African American population at its four outpatient dialysis facilities. We describe COVID-19 infection control measures implemented and clinical characteristics of patients with COVID-19 in the Emory Dialysis facilities.

**Methods:**

Implementation of COVID-19 infection procedures commenced in February 2020. Subsequently, COVID-19 preparedness assessments were conducted at each facility. Patients with COVID-19 from March 1–May 31, 2020 were included; with a follow-up period spanning March–June 30, 2020. Percentages of patients diagnosed with COVID-19 were calculated, and characteristics of COVID-19 patients were summarized as medians or percentage. Baseline characteristics of all patients receiving care at Emory Dialysis (i.e. Emory general dialysis population) were presented as medians and percentages.

**Results:**

Of 751 dialysis patients, 23 (3.1%) were diagnosed with COVID-19. The median age was 67.0 years and 13 patients (56.6%) were female. Eleven patients (47.8%) were residents of nursing homes. Nineteen patients (82.6%) required hospitalization and 6 patients (26.1%) died; the average number of days from a positive SARS-CoV-2 (COVID) test to death was 16.8 days (range 1–34). Two patients dialyzing at adjacent dialysis stations and a dialysis staff who cared for them, were diagnosed with COVID-19 in a time frame that may suggest transmission in the dialysis facility. In response, universal masking in the facility was implemented (prior to national guidelines recommending universal masking), infection control audits and re-trainings of PPE were also done to bolster infection control practices.

**Conclusion:**

We successfully implemented recommended COVID-19 infection control measures aimed at mitigating the spread of SARS-CoV-2. Most of the patients with COVID-19 required hospitalizations. Dialysis facilities should remain vigilant and monitor for possible transmission of COVID-19 in the facility.

## Introduction

In December 2019, the World Health Organization was notified of an outbreak of viral pneumonia of unknown etiology in Wuhan, China [1]. The disease was subsequently described as COVID-19 (coronavirus disease-2019), secondary to a novel coronavirus, SARS-CoV-2. On January 21, 2020, the first case of COVID-19 was reported in the United States in a patient in Washington State with recent travel from Wuhan [2]. The governor of Georgia announced the first two cases of COVID-19 in Georgia on March 2, 2020 and within a few weeks, there was evidence of community transmission in Atlanta, Georgia [3]. As of May 31, 2020, there were a total of 49,882 confirmed cases of COVID-19 and 2357 deaths across Georgia [4].

Persons at high risk of severe illness from COVID-19 include older patients (≥ 65 years), residents of nursing homes or long-term care facilities, persons of minority race/ethnicity, and persons with underlying conditions including diabetes, cardiac disease, pulmonary disease or asthma, severe obesity (BMI of 40 or higher), liver disease, immunocompromised conditions, and end-stage renal disease (ESRD) undergoing dialysis [5]. While data on the mortality rate of patients with ESRD receiving maintenance dialysis are limited, the first reported death from COVID-19 in the United States was a patient with multiple comorbidities including ESRD requiring maintenance hemodialysis at an outpatient dialysis facility [6]. In addition, Ma et al. reported a mortality rate of 16% (6 out of 37 patients) during a COVID-19 outbreak in a dialysis center in Wuhan, China [7].

The perceived susceptibility of patients receiving maintenance dialysis to COVID-19 infection led to nationwide efforts to provide guidance to dialysis facilities to safely dialyze patients with COVID-19 and prevent transmission of COVID-19 within the facility [8, 9]. Emory Dialysis rapidly devised and implemented its COVID-19 infection control plan. Emory Dialysis consists of four outpatient dialysis facilities across metropolitan Atlanta caring for approximately 750 patients requiring maintenance dialysis. The facilities are located in DeKalb and Fulton counties, which were the counties with the highest number of confirmed COVID-19 cases in Georgia in May 2020 [4]. Emory Dialysis serves an urban and predominantly African American patient population. We present implementation of COVID-19 infection control interventions in the Emory Dialysis facilities, describe the characteristics of patients with COVID-19 and highlight lessons learned managing patients on maintenance dialysis in the early stages of the COVID-19 pandemic.

## Methods

### Summary of infection control activities

**Fig. 1:**
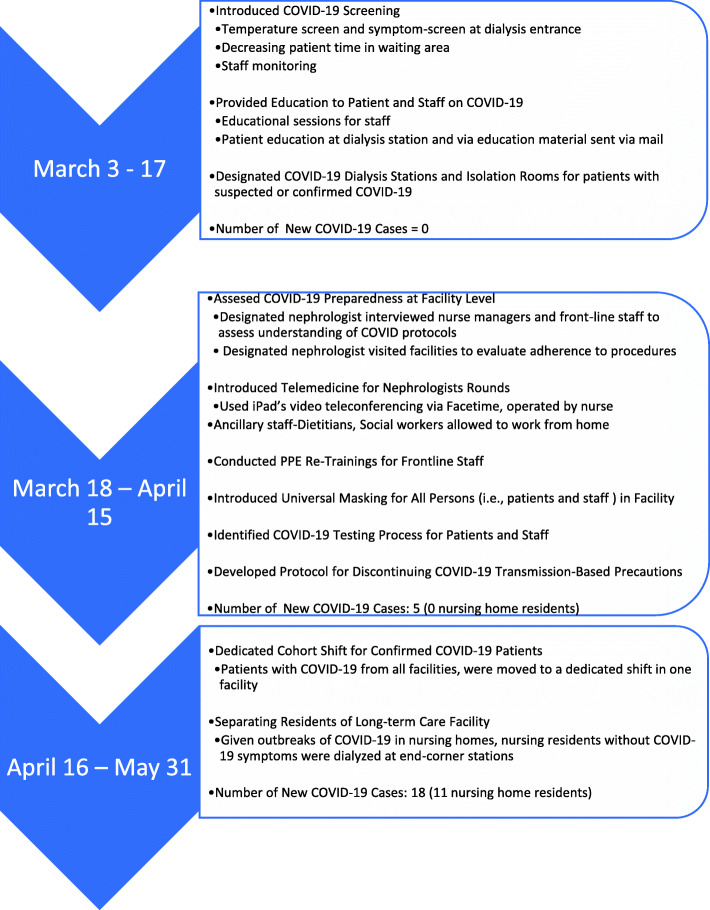
Timeline of Implementation of COVID-19 Infection Control Measures and Procedures at Emory Dialysis Facilities

Coordinated COVID-19 infection control procedures were introduced to all facilities. These infection control activities spanned three phases or time periods: 1) The initial preparedness activities occurred before there was evidence of SARS-CoV-2 community transmission and involved establishing procedures for screening, triaging and separating patients with symptoms of COVID-19, and monitoring staff for symptoms of COVID-19, 2) the second phase of activities occurred as there was evidence of community spread of COVID-19 in Georgia and involved additional measures to protect patients and staff including introduction of telemedicine, and universal masking for all persons in the facility, and 3) the third phase of activities involved managing a growing number of patients with confirmed COVID-19 in the dialysis facilities. [Fig. 1].

Initial Preparedness Activities.

Using COVID-19 resources from the Centers for Disease Control and Prevention (CDC) and professional societies, procedures and tools to identify patients with signs and symptoms of COVID-19 or exposure to a person with COVID-19 were developed. The triage station was moved to the facility’s entrance. The triage station was operated by a dialysis staff member, who conducted screening of all persons entering the facility including temperature checks and screening for symptoms and potential exposure to a person with COVID-19. Cough etiquette supplies including face masks for patients with respiratory symptoms or exposure to COVID-19 were placed in the triage station. The waiting room seating arrangement was modified to allow at least 6 ft of separation between the chairs. Efforts were made to decrease the length of time a patient spent in the waiting room and the number of patients in the waiting room at a given time. Patients with signs or symptoms of COVID-19 or potential exposures to COVID-19 were given facemasks and were asked to wait in their car (if clinically stable) or moved to a separate room. An isolation room (not being used as a hepatitis B surface antigen positive isolation room) was designated for dialysis of patients with suspected or confirmed COVID-19.

The facility management took inventory of their personal protective equipment (PPE) supply chain and aggressively sort PPE, while moving into contingency planning with PPE conservation strategies. Conservation strategies included extended use of N95 respirators by dialysis staff (instead of one-time use), extended wearing of dialysis staff treatment room gown, if not visually soiled (from one gown a day, to one gown a week) and extended use of face masks.

Patients who had symptoms of COVID-19 or exposure to a person with COVID-19 were considered Persons under Investigation (PUI). They were dialyzed in an isolation room or at end-station corner station (6 ft, in all directions from other patients), while wearing a mask at all times in the facility. The end-corner station was the dialysis bay with the least amount of patient and staff traffic. If testing results confirmed a diagnosis of COVID-19, the patient was dialyzed in an isolation room and usually occurred during the last shift of the day. Patients who were considered PUI or with confirmed COVID-19, did not spend time in the waiting room. They would come through a designated entrance to minimize exposure to other patients. As the number of patients with COVID-19 increased (> 6 patients), a dedicated COVID-19 shift was created at one of the dialysis facilities. Patients with COVID-19 from all the dialysis units were moved to the dedicated dialysis facility. Patients dialyzed in the COVID-19 dialysis shift also wore their masks during their time in the facility and were dialyzed in a specified bay and stations were separated by 6 ft (in all directions). Patients considered PUI remained at their respective dialysis facilities on their regular shifts and were dialyzed at an end-station corner station (of a designated bay) or in an isolation room pending results of the SARS CoV-2 test.

### Preparedness assessments

Following implementation of infection control procedures, a nephrologist with infection prevention experience, performed preparedness assessments at each facility using the Centers for Disease Control and Prevention (CDC)‘s COVID-19 preparedness assessment tool for dialysis facilities [10]. The assessment involved on-site meetings with the facility’s nurse manager and dialysis staff to review COVID-19 procedures and a walk-through of the facility to evaluate designated dialysis stations and isolation rooms for patients with suspected and confirmed COVID-19, and patient and staff screening and triage areas. Feedback was provided to the facility management with recommendations. Follow-up calls and facility visits were conducted to assess outstanding issues and evaluate adherence to procedures. In addition, following the first two COVID-19 cases, infection control procedures were reviewed, and frontline staff provided feedback that resulted in minor modifications to the procedures.

### Introduction of telemedicine to Dialysis rounds

Infection control measures were also instituted to limit nephrologists’ visits to the dialysis facility especially for those caring for patients with COVID-19 in the hospital setting. Nephrologists had the option of using a HIPAA-compliant version of the Zoom application for telemedicine videoconferencing for virtual rounds. This system was also used for monthly virtual rounds for patients on home therapies (peritoneal dialysis and home hemodialysis). Virtual rounds were performed in a multidisciplinary manner with patient, dietician, social worker, nurse, and nephrologist. In addition, the nephrologist was able to open the respective patient’s dialysis medical record and share their screen to review labs with the patient in real-time.

### Testing for SARS CoV-2

During the first two phases of infection control activities, testing of patients with suspected COVID-19 or exposure to persons with COVID-19 for SARS-CoV-2 was limited. Patients with symptoms were sent to the Emory Hospital emergency rooms for testing. To prevent overburdening the emergency room services and limit potential exposures to SARS CoV-2 in the emergency room, we explored other testing options. By the third phase, we had an identified an outpatient testing option. By this time, Emory Healthcare established a COVID-19 hotline that scheduled SARS CoV-2 PCR testing for staff and patients at an outpatient testing site. We worked with Emory Healthcare leadership to prioritize Emory Dialysis patients given the potential for transmission to other patients in the dialysis facility. Results of testing at the Emory COVID-19 testing sites was received within 48 to 72 h. Emory Dialysis also received testing results from other healthcare settings or via patient self-reporting when testing was done outside the Emory Healthcare system.

### Discontinuing COVID-19 transmission based precautions

Patients diagnosed COVID-19 remained on transmission-based precautions (i.e. isolation room or dedicated COVID-19 shift) until they met the following criteria: 1) afebrile for at least 72 h (without using a fever-reducing medication) and resolution of symptoms, and 2) 21 days had passed from onset of symptoms. We decided on 21 days instead of CDC’s recommended 10 days due to an abundance of caution and given the potential risk of prolonged viral shedding due to immune dysfunction seen in patients with advanced kidney disease.

### Data collection

To assess the clinical characteristics of patients who were diagnosed with COVID-19, data were abstracted from the facilities’ electronic medical records. Clinical care and laboratory data were collected per routine dialysis procedures and protocol. Emory Institutional Review Board approval was obtained, and informed consent was waived. A patient with confirmed diagnosis of COVID-19 was defined as a patient with a documented positive SARS-CoV-2 viral test (PCR or antigen). Patients with COVID-19 from March 1, 2020 through May 31, 2020 were included. The follow-up period spanned March– June 30, 2020, to ensure all patients had at least 30 days of follow-up from a positive SARS-CoV-2 test result. We report the baseline characteristics of all patients receiving care at Emory dialysis (i.e. Emory general dialysis population) for the period March1, 2020 through May 31, 2020.

### Statistical analysis

For the clinical characteristics of patients with COVID-19 and the general dialysis population, we performed descriptive statistics including counts, means, medians, interquartile ranges and percentages.

## Results

### Preparedness assessments

The first phase of COVID-19 Preparedness Assessments performed at each facility revealed all areas of preparedness checklist had been met except: 1) some of the designated stations for suspected or confirmed COVID-19 did not meet the requirement of a minimum of 6 ft from other dialysis stations (in all directions); 2) a local public health point of contact for COVID-19 had not been identified; 3) a non-hospital setting to refer patients and staff for SARS-CoV-2 testing had not been identified; 4) PPE re-trainings were underway; and 5) fit-testing for N95 respirators for dialysis staff was unavailable. On a two-week follow up visit, these outstanding areas had been appropriately addressed at all four facilities except for N95 respirator fit-testing. In some facilities, an isolation room (not being used for dialyzing patients who test positive for hepatitis B surface antigen) was designated for dialyzing patients with suspected or confirmed COVID-19. In another facility, a polycarbonate sheet was installed as a barrier between dialysis stations that were behind each other (with less than 6 ft of separation). A process of reporting COVID-19 cases to the Georgia Department of Public Health was established. Fit-testing for N95 respirators was unavailable due to shortage in fit-testing supplies in the country.

### Evaluating potential COVID-19 transmission within the facility

Emory Dialysis reviewed clinical characteristics of each new case of COVID-19 and assessed for signals of potential transmission in the facility. All COVID-19 cases were reported to the Georgia Department of Public Health. Following identification of a patient with COVID-19 at one of the dialysis facilities, there was concern for transmission within the facility. Patient 1 and Patient 2 dialyzed on the same shift and in the same bay (section) of the dialysis facility. Patient 1 had a known COVID-19 exposure at home but Patient 2’s exposure outside the dialysis facility is unknown. The timing of diagnosis of Patient 1 and Patient 2 was within a 5-day window. Approximately 10 days later, two patients (Patient 3 and Patient 4) at the same facility, dialyzing on different days of the week from Patient 1 and 2 were diagnosed with COVID-19. Patient 3 and Patient 4, dialyzed on the same shifts with their dialysis stations adjacent to each other, and were diagnosed with COVID-19 within 48-h of each other. It is unclear whether Patient 3 and 4 had interactions outside the facility. A dialysis staff who cared for Patient 3 and Patient 4 was also diagnosed with COVID-19 within a 10-day period of caring for both patients. Due to limited testing availability, testing of asymptomatic patients in the facility was not done to identify asymptomatic COVID-19 cases. In response to potential transmission within the facility, infection control education, infection control audits and PPE re-trainings were conducted to bolster infection control practices. Given asymptomatic COVID-19 and the fact that one of the patients who was asymptomatic at triage developed symptoms during the dialysis treatment, the decision was made to implement universal masking in the dialysis facility. All patients and staff were required to wear surgical masks throughout their time in the facility. A surgical mask was provided to patients on a weekly basis (PPE conservation strategy). Introduction of universal masking preceded CDC’s recommendations that all persons in dialysis facilities should wear face coverings/medical facemasks.

### Characteristics of Emory general dialysis population

Of the 751 patients followed at Emory Dialysis facilities from March through May 31, 2020, 619 (82.6%) were receiving in-center hemodialysis and 132 (17.6%) were on home dialysis (hemodialysis and peritoneal dialysis). The median age of the general Emory Dialysis patient population was 59 years (IQR, 49–69). Forty-eight percent were female, and 88% were black or African American. The median BMI was 26.7 (IQR 22.8–32.6). Among the patient’s receiving in-center hemodialysis approximately 30 patients (4.9%) were resident of nursing homes. Seventy-nine percent of patients had a diagnosis of hypertension, 53% had diabetes, and 49% had cardiac disease.

### Characteristics of patients with COVID-19

Twenty three Emory Dialysis patients (3.1%) were diagnosed with COVID-19 from March 21—May 31, 2020. Among the 23 patients diagnosed with COVID-19, 21 were receiving in-center hemodialysis and two were on peritoneal dialysis. [Table 1] The median age was 67.0 years (interquartile range [IQR] 63.2–70.3) and 13 patients (56.5%) were female. The median BMI was 27.3 (IQR, 24.4–30.2). Eleven patients (47.8%) were residents of nursing homes. Nineteen patients (82.6%) had a diagnosis of hypertension, 13 (56.5%) had diabetes, and 10 (43.5%) had cardiac disease. Nineteen patients (82.6%) required hospitalization and six patients (26.1%) died. Among the patients who died, the average number of days from a positive SARS-CoV-2 test to death was 16.8 days (range 1–34).

**Table 1:** Summary of characteristics of patients with confirmed COVID-19 and general dialysis population receiving dialysis at Emory Dialysis Facilities, March—May 2020

Characterstics	Patients with confirmed COVID-19 *n* = 23	Emory Dialysis general population^a^ *n* = 751
Median Age in years, (interquartile range)	67.0 (63.2–70.3)	59.0 (49–69)
Percent Female	56.5%	48.0%
Median BMI (interquartile range)	27.3 (24.4–30.2)	26.7 (22.8–32.6)
Race and Ethnicity		
Black, Non-Hispanic	78.3%	87.2%
White, Hispanic	13.0%	2.1%
White, Non-Hispanic	8.7%	7.9%
Other	0	2.8%
ESRD Etiology		
Hypertension	65.2%	55.0%
Diabetes Mellitus	21.7%	18.9%
Dialysis Modality		
In-center hemodialysis	91.3%	82.4%
Peritoneal dialysis	8.7%	16.0%
Comorbidities		
Diabetes Mellitus	56.5%	52.5%
Hypertension	82.6%	78.9%
Cardiac disease	43.5%	48.8%
Pulmonary disease	4.3%	10.5%
On ACEI/ARB	21.7%	31.3%
Potential Exposures to COVID-19		
Household member with confirmed COVID-19 diagnosis	17.4%	–
Nursing home resident	47.8%	4.9%
Outcome		
Hospitalization	82.6%	8.2%
Death	26.1%	–
Mean No. Days from Positive SARS-CoV-2 Test to Death (range)^b^	16.8 (1–34.0)	–

^a^Emory Dialysis general population includes all patients receiving dialysis care at Emory Dialysis including patients diagnosed with COVID-19 during the time period

^b^Among 6 deaths

## Discussion

The COVID-19 pandemic presented an immediate challenge to dialysis providers across the world to provide dialysis safely and prevent transmission of SARS-COV-2 in outpatient dialysis facilities [11]. There are published reports from single center dialysis facilities on their preparation and infection control plans in response to the pandemic [6, 12–14] as well as response and mitigations strategies in response to COVID-19 outbreaks in the dialysis facility [7, 15]. We present our preparedness efforts, challenges and successes in identifying and managing patients with COVID-19 in our dialysis facilities over a longer time period (12 weeks) in the United States. Emory Dialysis rapidly implemented procedures to screen patients for COVID-19, and separate and dialyze patients with suspected or confirmed COVID-19 from other patients in its facilities. The number of COVID-19 cases appear to be low and while horizontal transmission of COVID-may have occurred in one of the facilities, our vigilance and rapid response may have prevented a “full-blown” outbreak. While the findings presented here reflect data from the early stages of the COVID-19 pandemic, our successes in preventing or limiting SARS CoV-2 transmission in a dialysis facility that serves a vulnerable predominantly African American population is noteworthy. Lessons learned from our experienced are applicable to the infection control efforts in the current pandemic and can guide preparedness efforts for future pandemics.

**Fig. 2:**
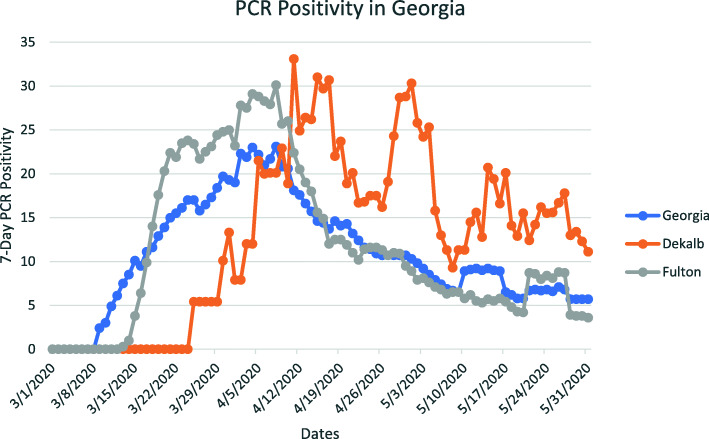
7-Day SARS CoV-2 PCR Positivity in Georgia and two counties in Georgia. Data from Georgia Department of health

We presented COVID-19 cases spanning March 1, 2020 through May 31, 2020. The 7-day SARS-CoV-2 PCR positivity rates for the state of Georgia, Dekalb county and Fulton county are shown in Fig. 2. In Fulton county, the PCR positivity rate was greater than 5% from March 16 to May 31, 2020, with the highest positivity rate of 30% on April 8, 2020. Similarly, Dekalb county 7-day SAR CoV-2 PCR positivity rate was greater than 5% from March 25, 2020 to May 31, 2020; the highest positivity rate of 33% on April 11, 2020. [Fig. 2] Notably, positivity rates are affected by the burden of SARS-Co-V-2 in a given community and amount of testing being done [16]. Our report spans a time period with limited SARS CoV-2 testing in Georgia. [Fig. 2] Further, state-wide pandemic measures were instated. On March 23, 2020 an order banning large gatherings (of > 10 persons) was issued and Shelter in Place was ordered from April 3 through April 30. The Shelter in Place order was extended for the medically fragile and elderly until June 12. These measures may have contributed to the low rate of COVID-19 cases seen at Emory Dialysis.

We found that patients with COVID-19 were older and there was a high percentage of nursing home residents. The majority (> 80%) of patients with COVID-19 required hospitalization. However, hospitalizations may not entirely reflect disease severity; in the early phases of the pandemic some patients who could not appropriately isolate at home or in a nursing home were hospitalized to prevent further community spread. Nursing homes experienced challenges isolating and separating patients with COVID-19 from other nursing home residents [17, 18].

Implementing recommended COVID-19 infection control measures pose a unique challenge in the outpatient dialysis setting. The open room layout of dialysis facilities, with dialysis stations less than 6 ft apart without a physical barrier between them, presents opportunities for transmission of SARS-CoV-2. In essence, “social distancing”, a recommended intervention to prevent transmission of SARS-CoV-2 may not be feasible in the outpatient dialysis facility [13, 14]. Dialysis facilities may not have an isolation room available (that is not being used for dialyzing a patient who is hepatitis B surface antigen positive) and are often operating at full patient capacity (no unassigned dialysis stations) [13] a challenge to separating patients with symptoms or signs of COVID-19 from other patients. Another potential obstacle to social distancing in this population is shared transportation used by a proportion of dialysis patients to get to their dialysis facility three times week. Further, dialysis staff usually care for multiple patients in a given shift and may serve as a vector for transmission of SARS-CoV-2 in the facility. Given all these considerations, Emory Dialysis created protocols and procedures to prevent transmission of SARS-CoV-2 in the facilities and to safely dialyze patients with suspected or confirmed COVID-19. While designated dialysis stations and isolation rooms for patients with suspected or confirmed COVID-19 differed across the facilities due to unique layouts and station/room availability, COVID-19 protocols and procedures were uniform across all facilities.

Creating a dedicated dialysis shift to cohort patients with confirmed COVID-19 dialysis that served all the Emory dialysis facilities allowed us to limit patient and staff exposure to SARS CoV-2. It also simplified logistical planning such as coordinating schedules for limited isolation rooms at each facility. However, it required staff (e.g. social worker and clinical managers) to coordinate with patients, transportation companies, hospitals, and long-term care facilities to ensure patients would transition to the dedicated shift. Patients were generally receptive of the changes in dialysis schedules, although one of the patients missed several dialysis treatment sessions. We initially limited movement of dialysis staff between facilities, but with the creation of an additional shift (the dedicated COVID-19 shift), staff from any of the four facilities could sign-up to work on that shift and received additional incentive pay.

Identifying a mechanism for SARS CoV-2 testing outside Emory’s Emergency room improved our ability to test symptomatic patients for SARS CoV-2. However, coordinating testing was challenging as it required testing off-site and coordination of transportation to the testing site, in some instances. At the time, CDC recommended either of two options for discontinuing transmission-based precautions for patients with COVID; a symptom-based strategy or a test-based strategy. We opted for a prolonged time and symptom-based strategy, as the test-based strategy required two negative tests separated by 24 h. We recognized that coordinating testing would be too burdensome to the patients and dialysis staff that arrange the testing.

Our experiences and lessons learned during the COVID-19 pandemic may inform infection control measures in the current pandemic and potentially, future pandemics. First, a multi-disciplinary approach was critical in implementing protocols including working effectively with frontline dialysis staff, nurse managers, social workers, transportation companies, nursing homes, hospitals, nephrologists and medical directors. Second, ensuring bi-directional flow of information and communication between staff, patients and leadership allowed Emory Dialysis to modify and improve COVID-19 protocols to meet patient and facilities’ needs. Third, engaging with CDC, the Georgia Department of Public Health and professional organizations such as the American Society of Nephrology which had several COVID-19 focused educational and communication forums were critical to managing patients in the pandemic given the evolving understanding of the COVID-19 pandemic. For example, using the list of COVID-19 cases by nursing home facility provided by the Georgia Department of Health, we were able to separate residents of nursing homes with ongoing COVID-19 outbreaks from other patients. Fourth, regular on-site visits by facility management and leadership to address concerns from frontline staff and assess adherence to protocols and procedures may improve staff buy-in. Fifth, infection prevention procedures should be incorporated into new interventions and workflows. For example, defining appropriate moments for disinfecting and cleaning iPad used for telemedicine rounds. Sixth, infection control audits and feedback should continue to occur as lapses may lead to a transmission of SARS-CoV-2 to patients and staff. Seventh, patient engagement and education are critical to the success of infectious control measures and needs to occur at regular intervals. Otherwise patients may fail to disclose signs or symptoms of COVID-19 to prevent disruption in their routine dialysis schedule or changes to their dialysis station. Finally, staying vigilant to signals of potential SARS-CoV-2 transmission within the facility and contract tracing will allow for early identification and mitigation of an outbreak.

There are some limitations to our report. Asymptomatic persons with COVID-19 account for a proportion of COVID-19 cases [15, 19, 20]. Therefore, the number of COVID-19 cases may be an underestimate of the burden of disease as the majority of testing for SARS-CoV-2 was in symptomatic patients or patients in high risk settings such as nursing homes. We are yet to formally explore the impact of our measures on clinical measures and outcomes and patient satisfaction. As the pandemic evolves and hopefully ends, we will perform an in-depth analysis of the impact of the pandemic on clinical outcomes and patient experiences. We received positive feedback from patients on virtual rounds (telemedicine); some patients appreciated increased time and privacy with the dietician. A formal and in-depth analysis of patient experiences would be help inform patient care. Infection control procedures during the pandemic has potentially increased dialysis staff workload. It is important to learn how this has impacted staff’s work experience to guide future workflow and staffing during outbreaks, pandemics and disasters.

## Conclusion

The unprecedented COVID-19 pandemic is a threat to a vulnerable dialysis population. Implementing and adhering to CDC’s recommended infection control measures in outpatient dialysis facilities is critical to protecting patients and staff. Hopefully, lessons learned from this pandemic will inform future designs of dialysis centers to better accommodate social distancing and optimize infection control. On-site testing for SARS-CoV-2 and other respiratory infections (such as influenza) to facilitate separating patients who are ill from other patients, augment strategies to cohort patients with the same respiratory illness, and enhance contact tracing is a critical intervention that should be considered in early planning stages of any pandemic. Providing support to dialysis facilities to safely conduct on-site testing should be considered in the current pandemic and in preparedness efforts for future pandemics. Promoting and improving vaccine uptake for influenza, pneumococcal vaccines, and COVID-19 vaccines is an additional infection control strategy. Emory Dialysis will continue to stay up-to-date on CDC’s COVID-19 guidance and explore innovative ways to promote the safety of our dialysis patients and staff.

## Data Availability

The datasets used and/or analyzed during the current study are available from the corresponding author on reasonable request.
